# Development of allele specific hybridization assay and computer aided tool for detection of genetic variations in folate pathway genes

**DOI:** 10.1186/1755-8166-7-S1-P19

**Published:** 2014-01-21

**Authors:** Ravishankara, Shreeshakala, S Padmalatha Rai, Kerthana Prasad, C Deepthi, PM Gopinath, K Satyamoorthy

**Affiliations:** 1 Division of Biotechnology, School of Life Sciences, Manipal University Manipal, India 576104; 2 School of Information Sciences, Manipal University Manipal, India 576104

## Background

Most current single nucleotide polymorphism (SNP) genotyping methods are still too slow and expensive for routine use in large association studies that requires hundreds or more SNPs in a large number of DNA samples and for diagnostic purpose. Clinical implementation and ultimate use of nucleic acid biomarkers necessitate development of pragmatic detection assays that deliver adequate sensitivities, mutation selectivity and high throughput capabilities in a rapid, robust and cost effective manner. In the present study we have developed a sensitive and cost effective genotyping technique with computer aided tool for allele discrimination and implemented it to genetic epidemiology study.

## Material and methods

Modified allele specific oligonucleotide hybridization assay with elimination of DNA isolation step for PCR and novel allele discrimination method was used to screen folate metabolism gene polymorphisms in acute lymphoblastic leukemia patients and normal individuals. The plasma homocysteine and global DNA methylation was performed by HPLC method.

## Results

The developed novel allele discrimination method along with the newly developed image analysis software increases the sensitivity and elimination of DNA isolation step for PCR reduces the cost of the genotyping technique. The average kappa value of all designed probes was > 0.81. The technique was successfully designed for both diagnosis and research purpose. The evaluation of effect of gene polymorphism on plasma folate, homocysteine level and global DNA methylation explored that the only MTHFR C677T gene polymorphism is associated with elevated homocysteine level in healthy young adults. The genotyping of 22 folate metabolism genes explored that only RFC1 80GA and RFC1 80AA genotypes were significantly associated with increased risk of Acute Lymphoblastic Leukemia (n=203) when compare to healthy individuals (n=245) in south Indian population.

## Conclusions

The developed visual based allele specific oligonucleotide hybridization assay will be useful in clinical diagnostic system where large scale screening is often necessary without any sophisticated detection systems. RFC1 80GA and RFC1 80AA genotypes were significantly associated with increased risk of acute lymphoblastic leukemia. In young adults, only MTHFR C677T polymorphism is associated with elevated homocysteine level.

